# Anthraquinone dyes decolorization capacity of anamorphic *Bjerkandera adusta* CCBAS 930 strain and its HRP-like negative mutants

**DOI:** 10.1007/s11274-014-1595-2

**Published:** 2014-01-11

**Authors:** Teresa Korniłłowicz-Kowalska, Kamila Rybczyńska

**Affiliations:** Department of Environmental Microbiology, Laboratory of Mycology, University of Life Sciences, Leszczyńskiego 7, 20-069 Lublin, Poland

**Keywords:** *Bjerkandera adusta*, Peroxidases, Laccase, Carminic acid, Poly R-478, RAPD

## Abstract

Cultures of the anamorphic fungus *Bjerkandera adusta* CCBAS 930 decolorizing, in stationary cultures, 0.01 % solutions of carminic acid and Poly R-478, were characterised by a strong increase in the activity of the horseradish peroxidase (HRP-like) and manganese-dependent peroxidase (MnP) at a low activity of lignin peroxidase. Genotypically modified mutants of *B. adusta* CCBAS 930: 930-5 and 930-14, with total or partial loss of decolorization capabilities relative to anthraquinonic dyes, showed inhibition of the activity of HRP-like peroxidase and MnP. Whereas, compared to the parental strain, in the mutant cultures there was an increase in the activity of lignin peroxidase and laccase. The paper presents a discussion of the role of the studied enzymatic activities in the process of decolorization of anthraquinonic dyes by the strain *B. adusta* CCBAS 930.

## Introduction

Dyes are substances used in various branches of industry (for the production of textiles, cosmetics, colour paper, pharmaceuticals, food etc.). Ineffectiveness of the process of dyeing causes that, depending on the type of dye applied, from 2 to 50 % of the dye used can find its way to wastewater (O’Neill et al. 1999). In the course of the process of dyeing, post-production wastewaters receive 5–20 % of acid dyes, 10 % of dispersive dyes, and 5–30 % of direct-effect dyes (Goncalves et al. 2000). Worldwide, annual losses of dyestuffs, accrued in the process of synthesis and application, amount to 15 %, which corresponds to 130 tons of dyestuffs per day, penetrating the environment (Maćkowiak and Urbaniak 2005). Jin et al. (2007) reported that, globally, annually, the amount of dyes entering the textile industry sewage is at least 280.000 tons. Due to the very large amounts of water used in the process of dyeing, at the level of 100 l per 1 kg of textiles dyed (Rodrigez Couto 2009), with daily production of 3 tons of textiles (Goncalves et al. 2000), only a single textile plant can generate 300.000 l/day of coloured post-production effluents.

Although many of the dyestuffs produced are compounds that are non-toxic or only weakly toxic to the environment (Goncalves et al. 2000), their introduction in waters, due to their dark colouring, limits the penetration of sunlight, inhibiting the process of photosynthesis, which reduces the concentration of oxygen and leads to a disturbance of the biological balance in aquatic ecosystems (Banat et al. 1996; Goncalves et al. 2000; Kuhad et al. 2004). Introduction of non-decolorized wastewaters into waters constitutes also a serious aesthetic problem. Colour is visible already at dye concentration of ca. 1 mg/l of water, while the concentration of those substances in the effluents from textile plants may reach even 300 mg/l (O’Neill et al. 1999). Factories manufacturing wool fabrics or polyester fibre produce effluents containing dyes in amounts of 10–50 mg/l (Goncalves et al. 2000). It is also a known fact that certain dyes, especially azo compounds and anthraquinonic dyes, are toxic for humans and animals and posses mutagenic, carcinogenic and co-carcinogenic properties (Cripps et al. 2005; Banat et al. 1996; Ali 2010). Those substances, via the food chain, penetrate and accumulate in the tissues of animals, especially of aquatic animals (Kuhad et al. 2004).

Methods applied so far for the purification of post-dyestuff wastes are mostly of the nature of chemical or physicochemical processes. This methods are usually costly and frequently do not ensure the desired effect of total decolorization (Fu and Viraraghavan 2001). Some of them involve the risk of formation of colourless toxic by-products, such as aromatic amines (Banat et al. 1996; Forgacs et al. 2004). Decolorization with the use of biological methods, involving microbial consortia of the activated sludge, also creates considerable problems due to the high resistance of dyes to biodegradation (Sugano et al. 2006) and to the toxic effect many of them have towards bacteria (Ali 2010). Moreover, the bio-decolorization of dyestuffs with bacterial participation may involve the creation of toxic products of biotransformation, aromatic amines in particular (Dawkar et al. 2009). This problem has not been signalled so far with relation to fungi, though mycoremediation progress more slowly than remediation with participation of bacteria (Ali 2010).

Among fungi, the most effective decolorizers of dyestuffs include the wood white rot fungi (Rodrigez Couto 2009). Those basidiomycetes produce oxidoreductases, that participate not only in the process of oxidative degradation of lignin but also of many other structurally related aromatic compounds, including a number of dyes: azo, anthraquinonic, heterocyclic, triphenylmethane (Kuhad et al. 2004). The process of decolorization of dyes takes place with the participation of peroxidases: lignin peroxidase (ligninase, LiP, EC 1.11.1.14), manganese peroxidase (MnP, EC 1.11.1.13), versatile peroxidase (VP, EC 1.11.1.16), and laccase (EC 1.10.3.2). In the various species of white rot fungi the biocatalysts of decolorization of dyes are various peroxidases and/or laccase. In the case of *Phanaerochaete chrysosporium*, thoroughly studied in this respect, LiP is responsible for decolorization (of azo, antraquinonic, triphenylmethane, heterocyclic dyes), while MnP does not display such properties (Ollikka et al. 1993; Wang et al. 2008). The participation of ligninase, but not manganese peroxidase, in the decolorization of azo and anthraquinonic dyes by *Trametes versicolor* ATCC 24725 was also demonstrated by Young and Yu (1997). Champagne and Ramsay (2005) revealed that in the decolorization of azo dyes by *T.* *versicolor* ATCC 20869 the key role is played not by ligninase, but by manganese-dependent peroxidase which, however, was inactive toward anthraquinonic dye Remazol Brillant Blue R (RBBR). That dye (RBBR) was decolorized faster than azo-dyes, from five- to ten-fold, by laccase of *T.* *versicolor* ATCC 20869 (Champagne and Ramsay 2005). Laccase of *Trametes* *versicolor* ATCC 48424 was also the primary biocatalyst in the decolorization of azo, anthraquinonic and indigo dyes (Wong and Yu 1999). This enzyme was also the only ligninolytic enzyme responsible for the decolorization anthraquinone and azo dyes by *Trametes trogii*—a new strain of white rot fungus isolated and identificated by Zeng et al. (2011). Versatile peroxidase, an enzyme with traits of LiP and MnP, catalyses the decolorization of various groups of dyes by fungi from the genus *Pleurotus*: *P. eryngii*, *P. ostreatus*, *P. pulmonarius*, and from the genus *Bjerkandera*: *Bjerkandera sp*. strain BOS 55, *Bjerkandera* sp. strain B33/3 and *B. adusta* (de Jong et al. 1992; Heinfing et al. 1997, 1998a, b, c, Master and Field 1998; Camarero et al. 1999; Cohen et al. 2002; Moreira et al. 2005, 2006, 2007). Among those white rot fungi, the broadest spectrum of decolorized dyes is characteristic of the strains of *Bjerkandera*, comprising azo, anthraquinonic, triphenylmethane and heterocyclic dyes (de Jong et al. 1992; Heinfing et al. 1997, 1998a, b, c; Master and Field 1998; Camarero et al. 1999; Moreira et al. 2005, 2006).

Korniłłowicz-Kowalska et al. (2006) described a new anamorphic strain of *Bjerkandera adusta*, CCBAS 930, with decolorization capabilities. *B. adusta* CCBAS 930 was isolated from soil with the use of, as the substrate, post-production biomass rich in daunomycin—a derivative of anthraquinone with red-brown colouring. Based on its phenotypic (micro- and macromorphological) traits, that strain was classified as *Geotrichum*-*like* (strain R59) as it produced conidia (arthrospores) of the *Geotrichum* type. However, it differed from *Geotrichum* by the presence of abundant, fluffy and hyalinic aerial mycelium. Analysis of internal transcribed sequences (ITS): ITS1 and ITS2 and of the 5,8S rDNA region revealed the species identity of the isolated conidial fungus among the *B. adusta* (Willd ex Fr)P. Karst (*Basidiomycota*) (Korniłłowicz-Kowalska et al. 2006). In stationary cultures, *B. adusta* CCBAS 930 effectively decolorizes various derivatives of anthraquinone: apart from daunomycin, also mono- and polyanthraquinonic dyes, as well as post-production lignin and humic acids (Belcarz et al. 2005; Korniłłowicz-Kowalska et al. 2006, 2008; Korniłłowicz-Kowalska and Rybczyńska 2010, 2012). In decolorized culture filtrates of the fungus a peroxidase with activity similar to that of horseradish peroxidase (HRP-like) was identified, which was manifested in the oxidation of o-dianisidine, a substrate typical of vegetable peroxidases, and laccase. The biosynthesis of laccase was observed only in the presence of humic acids (Belcarz et al. 2005). The studied strain of

*B. adusta* showed also the MnP activity only in cultures containing lignocellulose (Belcarz et al. 2005).

The aim of this study was to characterise activities of phenoloxidases of *B. adusta* CCBAS 930 in cultures containing model anthraquinonic dyes: carminic acid (Ac) and Poly R-478, with the use of mutants of the fungus.

## Materials and methods

### Strains of fungi

The research included the anamorphic parent strain *B. adusta* CCBAS 930 and its two phenotypic mutants: 930-5 and 930-14. The isolation from soil, identification, morphological characterisation and identification sequences of the gene fragment rRNA: ITS1, 5,8S rRNA and ITS2 of parental strain *B. adusta* CCBAS 930 are presented in the papers by Korniłłowicz-Kowalska et al. (2006). Identification sequences of the fungus have been submitted to GenBank with assigned Accession number AY 319191 and the culture was deposited in the Culture Collection of Basidiomycetes Prague, Czech Republic as *B. adusta* CCBAS 930. Induction and selection of mutants of *B. adusta* CCBAS 930 with modified ligninolytic activity after the application of *N*-methyl-*N*′-nitro-*N*-nitrosoguanidine (NTG) and UV radiation are described by Korniłłowicz-Kowalska and Iglik (2007), and Korniłłowicz-Kowalska and Rybczyńska (2010). The induction of mutants was conducted in 0.01 % solution of NTG and through 5–10-min UV irradiation (UV-C-200-280 nm) of mycelium homogenate. The selection of mutants was performed in the test of decolorization of 0.2 % lignin in Park and Robinson medium (Park and Robinson 1969) with 0.25 % glucose. From among seven phenotypic mutants (felting and discoloration of the mycelium) with varied effectiveness of decolorization of 0.2 % post-industrial lignin in agar medium (Korniłłowicz-Kowalska and Rybczyńska 2010), for examination in the present study those were selected that discoloured lignin the fastest: 930-5 obtained after treatment with NTG and 930-14 after two-stage mutagenesis with NTG and UV.

### Anthraquinonic dyes

Two anthraquinonic dyes manufactured by Sigma were applied: Ac and Poly R-478. Ac is a natural dye, used mainly in the food industry; its composition includes an aromatic system with the structure of anthraquinone, linked with a monosaccharide unit. Due to its structure, Ac demonstrate notable similarity to daunomycin—an anthracyclinic antibiotic degraded by the strain *B. adusta* CCBAS 930 (Korniłłowicz-Kowalska et al. 2006). The Poly R-478 dye is a synthetic dye with polymeric structure (polyanthraquinone). Due to their structure, both dyes are also used as indicators of ligninolytic activity. This results from the highly complex structure of lignin molecules, which enforces the need to apply indirect methods of estimation of ligninolytic activity. Among those, of key importance are the methods of decolorization of dyes with aromatic structures, such as anthraquinonic dyes: Remazol Brilliant Blue R (RBBR), Ac, and Poly R-478, Poly B-411 (Eguchi et al. 1994).

### Extraction of DNA and PCR-RAPD analysis

The isolation of genomic DNA of the parental strain *B. adusta* CCBAS930 and its two mutants: 930-5 and 930-14 was carried out with the use of 7-day mycelium from liquid stationary cultures (28 °C) in a maltose extract medium (malt extract—3 g, yeast extract—3 g, peptone—5 g, glucose—10 g, agar—20 g, H_2_O dest.—1 dm^3^). The fungus mycelium was separated from liquid phase using sterile filter paper and washed by sterile distilled water. The fresh mycelium was ground in a mortar with quartz sand and DNA was extracted with the use of DNA Plant mini Kit (Quigen, Inc. Valencia California).

The extracted DNA was subjected to RAPD-PCR analysis according to the method described by Rakariyatham (2006). Ten ten-nucleotide primers were used: OPN2: 5′-ACCAGGGCA-3′; OPN4: 5′-GACCGACCCA-3′; OPN5: 5′-ACTGAACGCC-3′; OPN6: 5′-GAGACGCACA-3′; OPN7: 5′-CAGCCCAGAG-3′; OPN11: 5′-TCGCCGCAAA-3′; OPN12: 5′-CACAGACACC-3′; OPN13: 5′-AGCGTCACTC-3′; OPN14: 5′-TCGTGCGGGT-3′; OPN16: 5′-AAGCGACCTG-3′ (Sigma, Poland). The RAPD reaction was prepared in sample volume of 25 μl with an addition of the suitable primer at concentration of 10 pmol; 20 ng of matrix DNA and 12.5 μl HotStarTaq Master Mix (Quiagen, Poland) (2.5 U HotStarTaq DNA polymerase, 0.2 mM deoxynucleoside triphosphates (dNPTs), 1.5 mM MgCl_2_, 1 × PCR buffer). The stages of the reaction of amplification, comprising initial denaturation at 95 °C (15 min) for HotStarTaq polymerase activation, 35 cycles: denaturation at 94 °C (1 min), primer annealing at 38 °C (1 min), elongation at 72 °C (2 min) and final elongation at 72 °C (10 min) were conducted in the Mastercycler personal (Eppendorf). The products of the PCR-RAPD reaction were separated electrophoretically in 1.5 % agarose gel with an addition of 10 μg/ml ethidium bromide in buffer 1 × TAE (40 mM Tris/acetate, 2 mM EDTA, pH 8) and run at 8 V/cm of gel, then visualised in UV light and photographed. Characterization of amplified products was designed for each RAPD marker based on the molecular size and primer used GelScan 2.0 software (Kucharczyk, Poland) with used DNA GeneRuler Ladder 100 bp (Fermentas, Poland). Similarities among fungal genotypes were deduced on the basis of the number of shared amplification products (Nei and Li 1979). All bands were numbered and marked as P (if the band is present) or A (in the absence of the band). The data for genetic similarity coefficients was analysed by CLUSTALX software and UPGMA algorithm (Jeanmougin et al. 1998).

### Culture conditions

The fungi (parental strain and the mutants) were cultured in 50 cm^3^ of liquid modified Park and Robinson medium (Park and Robinson 1969) containing, in g dm^−3^: NH_4_NO_3_ 0.1; MgSO_4_·7H_2_O 0.5; KH_2_PO_4_ 0.2; glucose 2.5 (instead of 0.7 g/dm^3^); pH 6. Dyes were added in such amounts as to achieve their final concentration of 0.01 %. The inoculum was constituted three circles of mycelium with ∅ = 1 cm, obtained from 7-day culture on glucose-potato agar medium (200 g/dm^3^ potato, 20 g/dm^3^ glucose, 20 g/dm^3^ agar). The control for decolorization and enzymatic activity was respectively non-inoculated medium with dyes and cultures of *B. adusta* CCBAS 930 and its mutants without dyes. The cultures and the control were maintained for 2 weeks at 28 °C, under stationary conditions.

### Enzyme assay

The enzyme activities were determined spectrophotometrically. Clear extracellular culture fluids were obtained through centrifuging of the cultures at 7000×*g* for 5 min HRP-like activity was estimated according to Maehly and Chance (1954) as modified by Malarczyk (1984), in the presence of 0.01 % o-dianisidine (ε_460nm_ = 11.3 M/cm) as substrate in 0.1 M acetate buffer with pH 5.5, in the presence of 0.1 mM H_2_O_2_. Manganese-dependent peroxidase (MnP) activity was measured through oxidation with 1 mM MnSO_4_ in 50 mM sodium malonate, pH 4.5, and in the presence of 0.2 mM H_2_O_2_ followed by determination of the Mn^+3^—malonic acid complex (ε_270nm_ = 11.59 M/cm) according to Wariishi et al. (1992). Lignin peroxidase (LiP) activity was assayed according to Tien and Kirk (1988) in the presence of 20 mM veratryl alcohol (ε _310nm_ = 9.3 M/cm) in 40 mM tartrate buffer, pH 3, in the presence of 0.4 mM H_2_O_2_. Laccase activity was estimated according to Leonowicz and Grzywnowicz (1981), in the presence of syringaldazine as the substrate (ε_525nm_ = 6.5 M/cm) in 0.1 M citrate–phosphate buffer, pH 5.

In all assays, one unit of enzymatic activity (U) was defined as the amount of enzyme that oxidized 1 μmol substrate per minute under defined condition and activities was reported as U/l.

### Decolorization activity

The decolorization activity was determined by measuring the lowering of absorbance: for Ac at A_495nm_ (maximum of absorbance), for Poly R-478—A_519nm_ (maximum of absorbance). The concentration of dyes in the solution was determined from a model curve plotted for Ac and for Poly R-478.

## Results

### RAPD analysis and genetic similarity

**Fig. 1:**
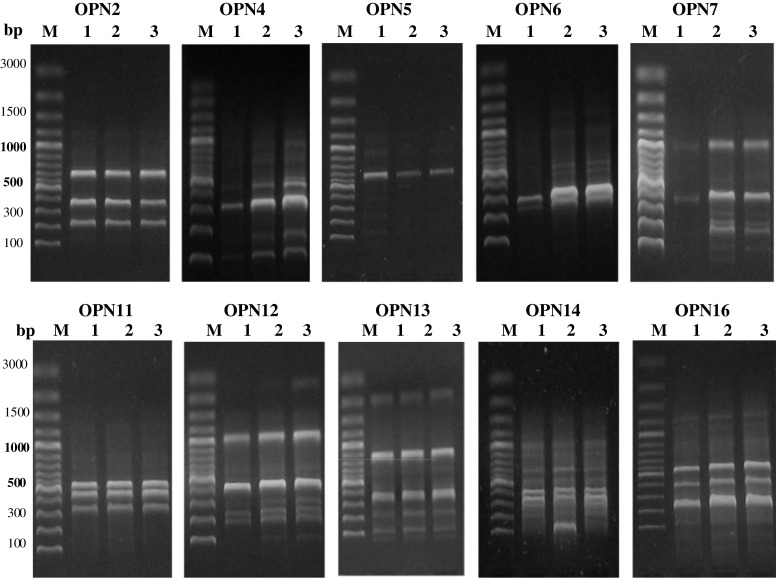
DNA fingerprint patterns using RAPD primers, *Lanes* *M*—100 bp marker, *1*—*B*. *adusta* CCBAS 930, 2—930-5, 3—930-14

Mutagenesis of *B. adusta* CCBAS 930 strain with use the use of NTG and UV radiation induced changes in the genetic material of the wild strain which affected the quality and quantity of the products of amplification and indicated differentiation of the genetic profile of parental strain and the mutants. The RAPD analysis applied, with the use of molecular typing of OPN starters, confirmed the existence of genotypic differences between the parental strain of *B. adusta* CCBAS930 and the phenotypic mutants studied: 930-5 and 930-14 (Fig. 1). Among the ten primers used, six (OPN4, OPN5, OPN6, OPN7, OPN12, OPN14) successfully discriminated the parental strain and to the mutants studied by amplifying polymorphic bands whereas four primers (OPN2, OPN11, OPN13, OPN16) not indicated molecular differentiation between amplifying bands. For six primers a total of 86 RAPD bands of good quality were produced, 39 of which were polymorphic (41 %). Percentage polymorphism in these genotypes ranged from 5.30 to 78.60 % (Table 1). The most significant differences in expression profile of different bands was indicated by primer OPN6 (78.60 %) (Fig. 1, Table 1). The genetic similarities analyses CLUSTALX, algorithm UPGMA) of three strains to proved that two mutants: 930-5 and 930-14 were similar in 45 and 43 % to parental strain whereas genetic similarity between mutants was 90 %. Based on genetic similarity analyses with the use chemical treatment revealed that NTG proved more effective as it change in genetic material of *B. adusta* CCBAS 930 strain indicated as compared to UV. Additional mutation with the use UV radiation induced only a few percentage changes in genetic profile of 930-14 mutant (Table 2).

**Table 1:** DNA polymorphism detected in mutant genotypes of *B. adusta* CCBAS 930

RAPD primer	Sequence (5′–3′)	Total no. of bands	Polymorphic bands	Polymorphism (%)
OPN4	GACCGACCCA	16	7	43.75
OPN5	ACTGAACGCC	8	5	62.50
OPN6	GAGACGCACA	14	11	78.60
OPN7	CAGCCCAGAG	14	8	57.15
OPN12	CACAGACACC	15	3	20.00
OPN14	TCGTGCGGGT	19	1	5.30

In the RAPD-PCR reaction with the use of OPN6 primer for all three strains amplification of a product with value of 300 bp was observed. In the case of mutants was present additional binding loci of OPN6 primer and characteristic of both mutants were 5 bands with values of 390, 550, 660, 1,000 and 1200 bp, whereas for parental strain characteristic was one additional band with values of 280 bp (Fig. 1). The RAPD reaction with the use of OPN5 primer for parental strain and both mutants indicated the product of amplification with values of 700 bp. In the case of parental strain of *B. adusta* CCBAS 930 were presented additional products: 150, 260, 360, 930 and 1100 bp which indicates the appearance of mutation at the site of OPN5 primer binding in the DNA of the both mutants.

**Table 2:** Genetic similarity coefficient matrix among *B. adusta* CCBAS 930 and its two mutants based on RAPD primers

Strain	*B. adusta* CCBAS 930 (parent strain)	930-5	930-14
*B. adusta* CCBAS 930 (parent strain)	1		
930-5	0.45	1	
930-14	0.43	0.90	1

Application of OPN4, OPN7, OPN12 and OPN14 primers in RAPD-PCR reaction additionally revealed genotypic differences between the mutants: 930-5 and 930-14. In the presence of OPN4 primer for parental strain and both mutants was observed products with values of 400, 300 and 100 bp. That band was only lightly marked in the DNA profile of the parental strain. In the case of the mutants, distinct differences were observed in the amount of the product formed, the highest intensity of amplification of that region being noted for mutant 930-14. Characteristic of both mutants were bands with values of 150 bp, 800 bp i 1000 bp. In the case of mutant 930-14 in the RAPD-PCR reaction additional product appeared, with values of 480 bp, indicating the polymorphism of DNA and additional binding loci of OPN4 primer. As a result of reaction of RAPD-PCR with use of OPN7 primer for parental strain *B. adusta* CCBAS 930 and mutants: 930-5 and 930-14 were amplified two products with values of 400 and 1000 bp. In the case of both mutants were characteristic four bands with values of range 100–280 bp, for 930-14 mutants was present additional binding loci of OPN7 primer and characteristic band with size of 80 bp.

In RAPD-PCR reaction with the use of OPN12 primer were amplified four products for all three strains with values of range 200–1,100 bp. In the case of both mutants were characteristic two bands with size of 100 and 500 bp and additional amplified product (1400 bp) in profile of 930-14 mutants. With the use of OPN14 primer for all three strain were amplified six product with the values of range 300–1000 bp. In the genetic profile of 930-14 mutants characteristic was present additional binding loci of OPN14 primer and band with size of 190 bp (Fig. 1).

### Decolorization of carminic acid and Poly R-478

**Fig. 2:**
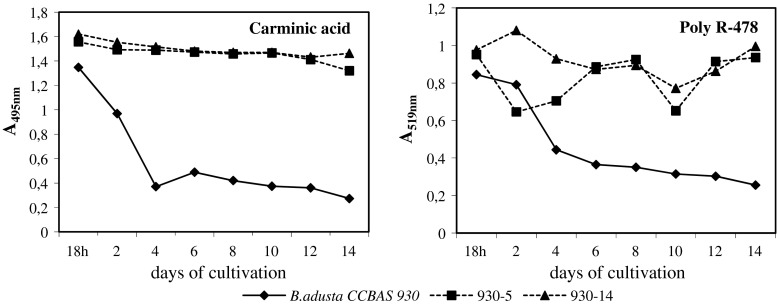
Decolorization of 0.01 % Ac and Poly R-478 by *B. adusta* CCBAS 930 strain and its mutants: 930-5 and 930-14 in stationary liquid cultures

Under static conditions, after 4 days of culturing the strain *B. adusta* CCBAS 930 removed more than 72 % of colouring caused by 0.01 % Ac. That corresponded to 76.82 % reduction of the concentration of that dye (Fig. 2, Table 3). A similar degree of decolorization (70 %) and reduction of concentration (77.28 %) of 0.01 % Poly R-478 was obtained only in 14-day cultures of that fungus. After 4 days of culturing of this strain removed only 47 % of colouring caused by 0.01 % Poly R-478, which corresponded to a reduction of concentration by 59.2 % (Fig. 2, Table 3). Visual brighting of the Ac solutions was observable from the 2nd day of growth of the fungus, and of Poly R-478—from the 4th day.

**Table 3:** Concentration of Ac and Poly R-478 in stationary liquid cultures of parental strain *B. adusta* CCBAS 930 and its mutants

Strain	Days of cultivation							
	18 h	2	4	6	8	10	12	14
Carminic acid (Ac)								
*B. adusta* CCBAS 930 (parent strain)	171.70 (±0.01)	123.20 (±0.05)	46.36 (±0.01)	61.49 (±0.01)	52.77 (±0.10)	46.74 (±0.02)	45.08 (±0.02)	33.79 (±0.01)
930-5	198.54 (±0.02)	190.21 (±0.04)	189.82 (±0.01)	187.77 (±0.01)	185.85 (±0.05)	186.87 (±0.02)	179.82 (±0.03)	168.03 (±0.01)
930-14	206.61 (±0.01)	198.07 (±0.01)	193.28 (±0.04)	188.67 (±0.01)	187.38 (±0.02)	187.38 (±0.02)	182.36 (±0.02)	186.36 (±0.01)
Poly R-478								
*B. adusta* CCBAS 930 (parent strain)	158.70 (±0.01)	148.30 (±0.06)	81.60 (±0.01)	66.40 (±0.02)	63.71 (±0.03)	56.79 (±0.01)	54.48 (± 0.01)	45.44 (±0.01)
930-5	179.29 (±0.01)	120.44 (±0.01)	131.79 (±0.01)	166.60 (±0.02)	174.10 (±0.03)	121.60 (±0.04)	171.98 (±0.01)	176.21 (±0.07)
930-14	183.90 (±0.01)	203.90 (±0.01)	174.86 (±0.01)	164.09 (±0.02)	168.13 (±0.03)	144,67 (±0.04)	162.17 (±0.02)	187.50 (±0.01)

It was found that the studied mutants of *B. adusta* CCBAS 930: 930-5 and 930-14, did not discolour Ac (Fig. 2). Whereas, a weak decolorization of Poly R-478 by strain 930-5 obtained through single-stage mutagenesis (NTG) was observed. The other mutant: 930-14, obtained from 2-stage mutagenesis (NTG and UV), did not cause any visually observable decolorization under the effect of Poly R-478.

### HRP-like activity

**Fig. 3:**
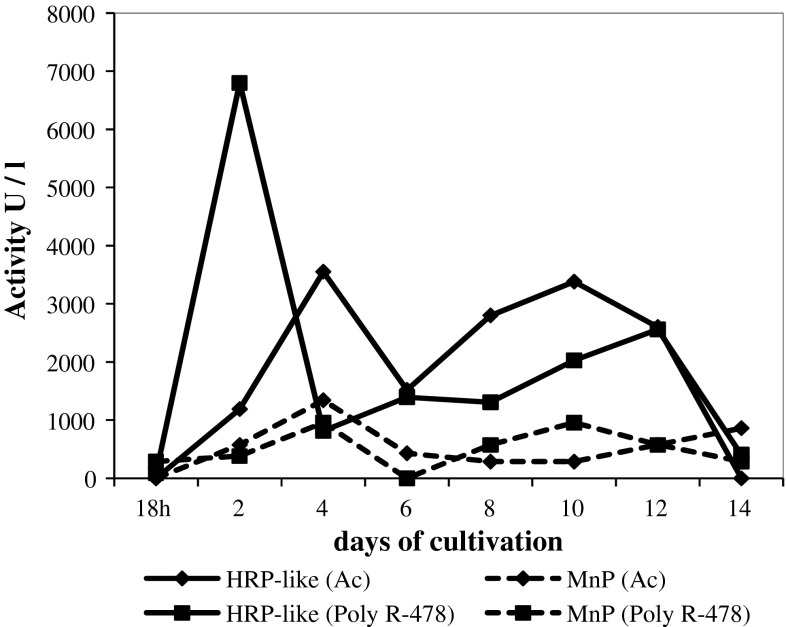
HRP-like and MnP activity in stationary cultures of parental strain *B. adusta* CCBAS 930 with 0.01 % Ac and Poly R-478

The HRP-like peroxidase activity was detected only in cultures of the parental strain CCBAS 930 growing in the medium containing anthraquinone dyes. The HRP-like activity was not detected in cultures of mutants growing in the medium containing Ac and Poly R-478. The HRP-like activity was not detected in cultures of parental strain and its mutants in the medium without dyes. It was higher in the presence of Ac than of Poly R-478. Activity of that enzyme in cultures of the parental strain supplemented with 0.01 % Ac was detected from the 2nd day of culturing with maximum on the 4th and again on the 10th days of culture, and amounted to 3,550 and 3,380 U/l, respectively. Towards the end of incubation extracellular HRP-like peroxidase activity in cultures of strain *B. adusta* CCBAS 930 decreased again (12-day cultures), up to complete inhibition in 14-day cultures (Fig. 3).

The dynamics of HRP-like peroxidase activity in stationary cultures of *B. adusta* CCBAS 930 in the medium with 0.01 % Poly R-478 differed from the dynamics of that enzyme activity in stationary cultures with 0.01 % Ac (Fig. 3). The highest activity was found already on the 2nd day of growth of the fungus. It was twice as high (6,769 U/l) as HRP-like peroxidase activity in stationary cultures with 0.01 % Ac. On subsequent days, the HRP-like peroxidase activity underwent a strong (eight-fold) reduction, to the level of 814 U/l after 4 days (while it increased in the cultures with Ac). Towards the end of culturing (12-day cultures) there was a recurrent, though weaker, increase in the activity of HRP-like peroxidase (2,560 U/l), followed by repression of synthesis of the enzyme (Fig. 3).

### MnP activity

The MnP activity of the parental strain was notably lower than HRP-like peroxidase activity and dependent on the dyes applied (Fig. 3). The MnP activity was not detected in cultures of all three strains without dyes. Higher activity of MnP was observed in the presence of Ac than of Poly R-478 (Fig. 3). With relation to both dyes the MnP activity was detected on the second day of growth of the fungus. The highest levels of MnP activity appeared after 4 days of culturing and amounted to 1,342 and 958 U/l, respectively (Fig. 3). In the cultures with Poly R-478, after 10 days of growth there appeared a second stimulation of MnP activity, equal to the first one (958 U/l) (Fig. 3). None of the mutants showed MnP activity in the medium without and supplemented with Ac. Whereas, the MnP activity in cultures of the mutants growing in the presence of Poly R-478 appeared only periodically, and it was significantly lower than that activity in the cultures of the parental strain (Table 4).

**Table 4:** Activity of manganese-dependent peroxidase (U/l) in stationary liquid cultures of *B. adusta* CCBAS 930 and its mutants with 0.01 % Poly R-478

Strain	Days of cultivation							
	18 h	2	4	6	8	10	12	14
*B. adusta* CCBAS 930 (parent strain)	287.00 (±0.12)	383.20 (±0.11)	958.80 (±0.09)	0	575.00 (±0.07)	958.50 (±0.10)	575.00 (±0.14)	287.60 (±0.11)
930-5	0	1,581.00 (±0,01)	0	0	0	575.00 (±0.01)	287.50 (±0.04)	0
930-14	383.30 (± 0.10)	287.50 (±0.02)	383.30 (±0.04)	0	0	287.50 (±0.01)	0	570.00 (±0.02)

### LiP activity

The lignin peroxidase was not detected in the cultures of parental strain *B. adusta* CCBAS 930 and its mutants without dyes. The LiP activity in the cultures of the parental strain and the mutants of *B. adusta* CCBAS 930 growing in media with Ac and Poly R-478 was detected throughout the period of the experiment. Compared to the efficiency of extracellular MnP, and especially of the HRP-like peroxidase, the levels of LiP activity was very low. In the course of the culturing, variations in the activity of that enzyme were generally slight, and somewhat stronger in the presence of Poly R-478 than on Ac (Table 5). Mutagenesis of *B. adusta* CCBAS 930 increased the efficiency of LiP in cultures with Ac, but not in those with Poly R-478 (Table 5).

**Table 5:** Activity of ligninase (U/l) in stationary liquid cultures of *B. adusta* CCBAS 930 and its mutants

Strain	Days of cultivation							
	18 h	2	4	6	8	10	12	14
Carminic acid (Ac)								
*B. adusta* CCBAS 930 (parent strain)	91.80 (±0.01)	91.80 (±0.01)	78.70 (±0.01)	87.50 (±0.01)	61.22 (±0.01)	78.70 (±0.01)	52.50 (±0.05)	78.70 (±0.02)
930-5	140.00 (±0.02)	140.00 (±0.02)	157.00 (±0.02)	297.00 (±0.07)	70.00 (±0.02)	219.00 (±0.01)	157.00 (±0.02)	149.00 (±0.05)
930-14	157.00 (±0.04)	122.00 (±0.01)	114.00 (±0.03)	315.00 (±0.09)	70.00 (±0.01)	175.00 (±0.02)	157.00 (±0.02)	236.00 (±0.02)
Poly R-478								
*B. adusta* CCBAS 930 (parent strain)	78.70 (±0.01)	70.00 (±0.01)	105.00 (±0.01)	26.24 (±0.01)	52.24 (±0.01)	26.24 (±0.01)	39.35 (±0.01)	26.24 (±0.01)
930-5	118.00 (±0.01)	118.00 (±0.04)	78.81 (±0.01)	78.70 (±0.01)	105.00 (±0.01)	105.00 (±0.01)	105.00 (±0.01)	0
930-14	114.00 (±0.01)	65.60 (±0.01)	87.46 (±0.01)	78.80 (±0.01)	91.83 (±0.01)	91.83 (±0.07)	91.80 (±0.01)	52.47 (±0.02)

### Laccase activity

It was found that the strain *B. adusta* CCBAS 930 and both of its mutants did not synthesise laccase (Lac) in control (cultures without dyes) and in cultures with Ac. Whereas, the enzyme was detected in cultures of those strains growing in the presence of Poly R-478 (Table 6).

**Table 6:** Activity of laccase (U/l) in stationary liquid cultures of *B. adusta* CCBAS 930 and its mutants with 0.01 % Poly R-478

Strain	Days of cultivation						
	2	4	6	8	10	12	14
*B. adusta* CCBAS 930 (parent strain)	0	0	0	0	0	251.30 (±0.09)	102.60 (±0.07)
930-5	223.10^a^ (±0.10)	307.70 (±0.04)	130.80 (±0.02)	377.00 (±0.03)	200.00 (±0.01)	646.20 (±0.01)	123.10 (±0.01)
930-14	61.54 (±0.01)	138.50 (±0.09)	0	46.15 (±0.01)	97.44 (±0.05)	338.50 (±0.11)	282.10 (±0.08)

Explanations: ^a^−58.85 (±0.01) after 18 h

Single-stage mutagenesis (with the use of chemical reagent NTG) of strain CCBAS 930 increased the biosynthesis of laccase in cultures with Poly R-478. It was showed that the parental strain synthesised laccase only on the 12th and 14th days of culturing, while the mutants did it throughout (930-5) or during most of (930-14) the period of incubation with that dyes.

In the cultures of all strains the highest laccase activity was observed after 12 days of growth Table 6

## Discussion

Mutagenesis with the use of nitrosoguanidine (NTG) and UV radiation induced changes in DNA of strain *B. adusta* CCBAS 930 and modified its decolorization properties.

The RAPD-PCR method is used for the purposes of identification of genetic differentiation between a wild strain and its mutants obtained through the effect of chemical and physical factors (Lee et al. 2000; Rakariyatham 2006; Shafique et al. 2009, 2011). As follows from our research, the application of the OPN4 starter in the RAPD-PCR reaction generated the appearance of various products of amplification for all three research strains, as a result of which different DNA profiles were obtained. It was found that the mutants obtained through single-stage (NTG) and two-stage (NTG, UV) mutagenesis, respectively, differed genetically from each other and as compared to the parental strain. The greatest polymorphism was characteristic of mutant 930-14, obtained as a result of two-stage mutagenesis. Shafique et al. (2009, 2011) also demonstrated greater genetic differentiation in comparison with the wild-type strain, of mutants of *A. tenuissima* FCBP-252 and *T. viride* FCBP obtained after UV irradiation as compared to mutants produced through the effect of chemical factors alone.

Chemical and physical factors generating genotypic changes in the mycelium are used, among others, for the purpose of inducing overproduction of enzymes with industrial importance. As an example, mutagenesis of such fungi as *Pleurotus ostreatus* (Lee et al. 2000), *Alternaria tenuissima* (Shafique et al. 2009), *Trichoderma viride* (Shafique et al. 2011), *Aspergillus* sp. (Rakariyatham 2006) and *Penicillium purpurogenum* (Sharma et al. 2005) caused stimulation of biosynthesis of α-amylase, cellulase, myrosinase and inulinase. On the other hand, UV radiation inhibited the ligninolytic abilities of the mutated strain *Phanerochaete chrysosporium* ME446 towards lignin and structurally related compounds (Boominathan et al. 1990). As follows from our earlier study, mutants of *B. adusta* CCBAS 930 (=R59): 930-5 (R59-5) and 930-14 (R59-14) decolorized post-industrial lignin more effectively than the parental strain (Korniłłowicz-Kowalska and Rybczyńska 2010). Whereas, they showed a decrease (930-5) or total loss (930-14) of decolorization activity in relation to anthraquinonic dyes.

Our research demonstrated that the parental strain of *B. adusta* CCBAS930, during growth in stationary cultures containing Ac and Poly R-478, prove four different extracellular phenoloxidases activities: HRP-like peroxidase, MnP, LiP and laccase. The decolorization of 0.01 % solutions of both dyes was coupled only with an increase in the activity of HRP-like peroxidase and of MnP. The efficiency of MnP (at maximum of its activity) was, however, from two- (in the presence of Ac) to four-fold (in the presence of Poly R-478) lower as compared with HRP-like peroxidase. The process of decolorization of the studied dyes by CCBAS 930 strain was not accompanied by changes in the activity of lignin peroxidase which was very low throughout the period of culturing. Laccase activity was detected only towards the end of the culturing, and only in the presence of Poly R-478. However, induction of that enzyme did not contribute to the decolorization of the remaining content (ca. 30 %) of that dyes. The relation between decolorization of anthraquinonic dyes and the HRP-like peroxidase activity of *B. adusta* CCBAS 930 was demonstrated also in our previous publication (Korniłłowicz-Kowalska and Rybczyńska 2012). An increase in the activity of peroxidase oxidizing o-dianisidine took place also during the decolorization of other derivatives of anthraquinone by *B. adusta* CCBAS 930: daunomycin, alkaline lignin and humic acids (Korniłłowicz-Kowalska et al. 2006, 2008).

The existence of a connection with the activity of horeseradish-type (HRP) and MnP peroxidases and the decolorization of anthraquinonic dyes by the strain

*B. adusta* CCBAS 930 is also indicated by the study of mutants of the fungus. It was demonstrated that the loss of decolorization abilities of the mutants towards Ac was coupled with a lack of HRP-like peroxidase and MnP activity. Mutant 930-5, that less effective decolorized Poly R-478, was also characterised by inability to produce HRP-like peroxidase, but it did produce MnP, though in small amounts. Whereas, the study excluded the participation of laccase and lignin peroxidase in decolorization of both dyes by *B. adusta* CCBAS 930, as the stimulation of the activity of those enzymes in the cultures of 930-5 and 930-14 did not contribute to the decolorization of the culture fluids.

It was found that, in terms of the phenoloxidase activity, strain *B. adusta* CCBAS 930 shows similarity to the strain *Irpex lacteus* KR35 W studied by Shin (2004). That author demonstrated a correlation between decolorization of the dye effluent (containing various dyes) and the activity of peroxidase oxidising o-dianisidine (earlier referred to as non-specific peroxidase—NsP), with a probable participation of MnP but not of LiP (very low level of activity, precluding its contribution in decolorization) and laccase (lack of activity during decolorization) synthesised by that white rot fungus.

The research were demonstrate that the range of phenoloxidase activities, the strain

*B. adusta* CCBAS 930 is similar primarily to other strains of *Bjerkandera,* such as *Bjerkandera* sp. strain B33/3 (Moreira et al. 2001). Moreira et al. (2001) demonstrated that *Bjerkandera* sp. strain B33/3, during decolorization of dyes Poly R-478 and Remazol Brilliant Blue R (RBBR), is also characterised by three different peroxidase activities: the activity of substrate-nonspecific peroxidase (non-dependent with manganese), MnP and LiP. In view of our own research, however, the similarity of the strain *B. adusta* CCBAS 930 and the strain *B. adusta* Dec 1, being the subject of long-term research (since 1995) by Japanese researchers: Kim et al. 1995; Kim and Shoda 1999; Sugano et al. 2006, 2009; Sugano 2009; Yoshida et al. 2011, should be considered as particularly interesting. Both of those strains display high decolorization activity towards derivatives of anthraquinone, and both are anamorphic stadia of *B. adusta*. Initially, the strain

*B. adusta* Dec 1 (Kim et al. 1995) was also classified as a mould fungus from the genus *Geotrichum* (*Geotrichum candidum* Dec 1). Subsequently, based on molecular criteria (region ITS and 5,8 s rDNA), it was included among the *Basidiomycota* as *Thanatephorus cucumeris* Dec 1 (Sugano et al. 2006) and next, after re-identification, as *B. adusta* Dec 1 (Sugano 2009; Yoshida et al. 2011). In turn, analysis of the internal trascribed sequences of *B. adusta* R59 strain, initially classified as *Geotrichum*–like R59, comprising ITS1, ITS2 and 5,8S of gene rRNA, which was described in detail in a paper by Korniłłowicz-Kowalska et al. (2006), yielded 2 sequences (ca. 620 base pairs), whose identical fragment, after comparison with sequences from the GenBank, corresponded both to *Thanatephorus cucumeris* (*Basidiomycota, Ceratobasidiales*) and *B. adusta* (*Basidiomycota*, *Polyporales*). Due to the fact that *T. cucumeris* (which is a teleomorph of *Rhizoctonia solani*) does not produce any conidial spores, while *B. adusta* produces conidia of the type of arthrospores, the studied strain CCBAS 930 was finally classified as an anamorphic stadium of *Bjerkandera adusta*.

The physiological similarity of the two strains: *B. adusta* CCBAS 930 and *B. adusta* Dec 1, is related with their specialisation in enzymatic decolorization of derivatives of anthraquinone—a process in which the key role is played by peroxidases of those fungi. Sugano (2009) reports that the peroxidase of *B. adusta* Dec 1, participating in the process of decolorization of anthraquinonic dyes, represents a new family of haemo-peroxidases—DyP peroxidase. Purified preparations of the enzyme showed greater decolorization activity towards anthraquinonic dyes than with relation to azo-dyes and other phenolic compounds (Sugano 2009). Although in the case of strain *B. adusta* CCBAS 930 the object of research were only crude enzymatic preparations (non-purified culture fluids), their activity, estimated on the basis of the rate of decolorization, was also the highest with relation to derivatives of anthraquinone: daunomycin and anthraquinonic dyestuffs (Ac, remazol brilliant blue, Poly R-478) (Belcarz et al. 2005; Korniłłowicz-Kowalska et al. 2006, 2008; Kornillowicz-Kowalska and Rybczynska 2012). Therefore, there is a possibility that strain *B. adusta* CCBAS930, in the presence of anthraquinonic dyes, produces a similar type of peroxidase as that produced by the strain *B. adusta* Dec 1. To prove whether this in fact is true, and whether the enzyme can be used in the decolorization and detoxification of post-industrial effluents containing anthraquinonic dyes or cytostatics (daunomycin), further studies are required, comprising the isolation, characterisation of properties and estimation of application possibilities of purified preparations of peroxidase (peroxidases) of the investigated strain.

It is also necessary to optimize decolorization anthraquinone dyes by strain of *B. adusta* CCBAS 930 including empirical modeling technique. In is regard of key importance are Response Surface Methodology. There are statistically designed experimental models, which allow for optimization of the culture studies using biological decolorization (Sharma et al. 2009; Taveres et al. 2009; Mohammadian Fazil et al. 2010; Papadopoulou et al. 2013). In case of white rot fungi such as

*B. adusta* CCBAS 930 decolorization process occurs by co-metabolism. With respect to the anthraquinone dyes decolorization by white rot fungi, the essential parameters for controlling the course of decolorization should include the concentration of the dye, a source of C and N and their concentrations, pH medium and temperature of incubation.
